# Prevalence and correlates of undiagnosed hypertension among staff of a Nigerian university community

**DOI:** 10.11604/pamj.2022.42.80.26464

**Published:** 2022-05-31

**Authors:** Babangida Shehu Bappah, Aishat Shittu, Jibrin Sammani Usman, Jibril Mohammed Nuhu, Farida Garba Sumaila, Aisha Ahmad Ahmad, Jibril Mohammed

**Affiliations:** 1 Department of Physiotherapy, Federal Medical Center, Azare, Nigeria,; 2 Department of Physiotherapy, Bayero University Kano, Kano, Nigeria

**Keywords:** Hypertension, prevalence, predictors, university community, undiagnosed

## Abstract

**Introduction:**

the prevalence of hypertension in Nigeria is high, with a considerable proportion of it being undiagnosed. Nevertheless, early identification of influencing variables for hypertension in different population groups is important for several reasons. This study aimed to determine the prevalence and correlates of undiagnosed hypertension among staff of a university community in Nigeria.

**Methods:**

a purposive sample of 281 staff of Bauchi State University, Gadau, Nigeria, fulfilled the inclusion criteria and were enrolled in this cross-sectional study. Demographics, blood pressure, height, weight, socioeconomic status and physical activity were measured. Diagnosis of hypertension was defined based on a systolic and diastolic blood pressure of ≥140 mmHg and ≥90 mmHg, respectively. Data obtained was analysed descriptively, and by means of Chi-square, univariate and multivariate statistics using SPSS v24 software.

**Results:**

the mean age and body mass index (BMI) of the participants was 34.5 years and 23.1 ± 5.17 Kg/m^2^, respectively. The prevalence rate of undiagnosed hypertension was 27.8%. Normotensives significantly differed from participants with undiagnosed hypertension in most of the clinical and demographic variables (p<0.05). Univariate and multivariate analyses revealed that a positive family history of hypertension had the highest odds of having undiagnosed hypertension (aOR: 0.833, 95%CI: 16.55-432.87, p= 0.000). Next, a higher BMI score (aOR: 0.425, 95%CI: 0.085-0.447, p= 0.000), male gender (aOR: 0.451, 95%CI: 0.141-0.829, p= 0.018), job cadre (aOR: 0.515, 95%CI: 0.073-0.550, p= 0.002) and low physical activity level (aOR: 0.572, 95%CI: 5.296-49.777, p=0.000) were other factors with about 50% odds for having undiagnosed hypertension among the participants. Lastly, smoking status and socioeconomic status of the participants were not significantly associated with undiagnosed hypertension (p>0.05).

**Conclusion:**

a high percentage of the studied population have undiagnosed hypertension that is mainly associated with non-modifiable (especially positive family history) and a few modifiable risk factors. These variables can be used for early identification and in designing appropriate preventive strategies.

## Introduction

Hypertension is a chronic non-communicable disease that affects people of all age groups, sex and socio-economic classes [1,2]. Despite the considerable progress that has been made in the prevention, detection, treatment and control of hypertension over the years, the disease remains a major cause of global morbidity and mortality, and of significant public health importance [3,4]. Hypertension is known to increase the risk of stroke, myocardial infarction, congestive heart failure, sudden cardiac death, peripheral vascular disease and renal insufficiency [5-8]. Furthermore, the onset of hypertension has been linked to a number of risk factors such as ageing, obesity, sedentary behaviour, family history of hypertension, African ancestry, smoking, use of contraceptive pills and excess intake of alcohol and salt [9-11].

The prevalence of undiagnosed hypertension has been reported to vary among different population groups/geographical locations [8,12], occupational endeavours [13], gender [14] and socioeconomic groups [2,8]. In sub-Saharan Africa, the burden of hypertension has been on the increase over the past few decades. Ataklte *et al*. (2015) [15] in a recent meta-analysis reported that a large proportion of individuals with hypertension remains undiagnosed, untreated, or inadequately treated, contributing to the rising burden of cardiovascular disease in the region. They further concluded that the prevalence of hypertension is high, and that hypertension awareness, treatment, and control remains low.

In West Africa, the prevalence of hypertension range from around 12% among physically active population, and up to 68% among those that are sedentary [16]. In Nigeria, the prevalence of hypertension has a much narrower range of between 12% and 36.8% [17-20]. However, the prevalence of undiagnosed hypertension among different population groups in Nigeria still requires further attention and intervention due to paucity of data. Already, increasing age, high BMI and following long working years have already been identified as major correlates of undiagnosed hypertension among employees of the tertiary hospital in Nigeria [19]. Nevertheless, additional studies may offer further understanding, planning and guide public health intervention strategies. Moreover, there is a need to further explore other socio-physical outcomes that could potentially correlate with undiagnosed hypertension in order to be able to adequately focus on preventive strategies.

We hypothesized that socio-physical correlates including demographic variables, educational attainment, job cadre, socioeconomic status, and self-reported physical activity levels could be used for forecasting the presence of undiagnosed hypertension in a specified population. Therefore, this study aimed to investigate the prevalence and correlates of undiagnosed hypertension among the staff of Bauchi State University, Gadau, Nigeria.

## Methods

**Study design and setting:** the design for this study was a cross-sectional study of staff of Bauchi State University, Gadau, Nigeria. The staff were recruited from the administrative units and across the six faculties of the university based on their availability and willingness to participate in the study. Data collection was done at their place of work (offices) between November 2019 and February 2020.

**Study population:** the population of the study comprised all staff of Bauchi State University, Gadau, Nigeria, who were not previously diagnosed or being managed for hypertension. The sample size was calculated using the formula presented by Daniel (1999) for estimating an adequate sample size as shown below [20]:


$$n=\frac{Z^{2}P(1-P)}{d^{2}}$$

Where n = sample size; Z = Z statistic for a level of confidence, at 95% confidence interval, Z = 1.96; P = expected prevalence or proportion which is estimated from a previous study by Addo *et al*. (2007) as 0.22 [21]; d = precision (at precision of 5%, d = 0.05).


$$n=\frac{1.96^{2}\times 0.22\times (1-0.22)}{0.05^{2}}=269$$

After estimating the sample size required, a total of 281 members of academic and non-academic staff of the university were recruited to participate using purposive sampling technique from the administrative units and across the six faculties of the university. The inclusion criteria of the study are as follows: i) Male and female full-time staff/employees of the Bauchi State University, Gadau; ii) aged between 18 to 65 years, and; iii) apparently healthy individuals. Participants who were pregnant (women), had a prior diagnosis of hypertension, had previous and current use of anti-hypertensive medications, and/or has physical disabilities, were excluded as highlighted in a similar past study [22].

**Data collection:** all relevant permissions from the university management, units and faculties were obtained prior to starting data collection. The participants were approached at their places of work to participate in the study. The objectives and significance of the study were explained at this stage. Thereafter, each potential participant was screened for eligibility based on the inclusion and exclusion criteria. Those who were potentially eligible to participate were then required to sign an informed consent. After obtaining consent from the participants, their biodata, blood pressure, height, weight and physical activity level were assessed or recorded using standardized procedures, accordingly.

**Definitions:** blood pressure of the participants was measured in the right arm after at least 15 minutes of rest and while participants were sitting down. The cuff (about 12.5 cm wide) was applied evenly and snugly around the bare arm, with the lower edge 2.5 cm above the antecubital fossa. The participants must not have eaten, ingested alcoholic drinks, smoked tobacco, or engaged in exercise training for at least 30 minutes before the measurements. Measurements were taken at least two times and the mean of two separate readings was determined after an interval of 10 mins and recorded to the nearest mmHg. Undiagnosed hypertension was defined if systolic blood pressure (SBP) was 140 mmHg and above or diastolic blood pressure (DBP) 90 mmHg. Blood pressure was classified according to the Seventh Joint National Committee criteria [23].

The physical activity level of the participants was determined by the Global Physical Activity Questionnaire (GPAQ) developed by the World Health Organization (WHO, 2012) for physical activity surveillance [24]. This is a 16-item questionnaire that is simple to administer and collects information on physical activity participation in three settings (or domains) as well as sedentary behaviour. The domains are: i) Activity at work; ii) travel to and from places and; iii) recreational activities. The GPAQ was administered by the researcher via face-to-face administration. The duration and frequency of physical activity (min/day) participation in the three domains (activity at work, travel to and from places, and recreational activities) over a typical week was recorded. Activities were classified as either: i) vigorous (8 metabolic equivalent tasks [METs]), and; ii) moderate (4 METs) intensities, and also by means of a summary estimate of the moderate to vigorous physical activity (MVPA) intensities, which was calculated by combining the activity score of both moderate- and vigorous-intensity activity for each work and recreational activity domain. The participants were then classified into two activity intensity categories (active and sedentary) according to their total physical activity per week (MET-minute per week) based on the GPAQ guidelines [25].

The participant´s socioeconomic status was assessed using a modified version of the Pittsburgh Cold Study 3 (PCS3) socioeconomic status questionnaire. The questionnaire takes into account measures that included traditional socioeconomic indices, such as educational attainment and household income (in US dollars), as well as less traditional indicators of social standing including home size and frequency of out-of-town vacationing. The modification made include: changing the currency name and value from US dollar to naira and adjusting its rates based on the socioeconomic realities of Nigeria at the moment. The items that were not too relevant in our settings such as: i) Out-of-town vacationing and; ii) newspaper delivery was removed. The questionnaire has a total score of 64 points. A score of 42.6 and above is considered a high socioeconomic status. A score of 21.3 to 42.5 is considered moderate socioeconomic status. A score less than 21.3 is considered low socioeconomic status [26].

The body mass index (BMI) was calculated as weight in kilograms divided by the square of the height in meters. The Centers for Disease Control and Prevention (CDC) classification of BMI was used to grade BMI values obtained from the height and weight as follows; underweight (<18.5 kg/m^2^), normal weight (18.5 to 24.9 kg/m^2^), overweight (25 to 29.9 kg/m^2^) and obesity (>30kg/m^2^) [27].

**Statistical analysis:** normality of the data was initially evaluated using Shapiro-Wilk´s test and also by manual inspection of histograms and QQ plots. Thereafter, descriptive statistics of mean, percentage, frequency and standard deviation were used to summarize the sociodemographic and clinical characteristics of the participants, and the prevalence of undiagnosed hypertension. Mann Whitney U test was employed to compare the continuous (non-normally distributed) variables between participants with undiagnosed hypertension and normotensives. Chi-square test was utilized to test the association between the categorical (independent) variables between participants with undiagnosed hypertension and normotensives. Furthermore, univariate and multivariate logistic regression analyses were conducted to explore the significant correlates among the independent variables for undiagnosed hypertension (expressed in categorical terms [yes or no]). Only independent variables that were significant during the univariate analyses were included in the model of the multivariate analyses. The threshold for the univariate probability (p) value for inclusion in the multivariable model was set at 0.2 in order to consider several potential confounders in the analyses. Alpha probability level was significant at 0.05.

**Ethical considerations:** prior to the commencement of the study, ethical approval was sought for and obtained from the Bauchi State University Research Ethics Committee, Bauchi, Nigeria (BASUG/FBMS/REC/ VOL. 2/001).

## Results

**Figure 1:**
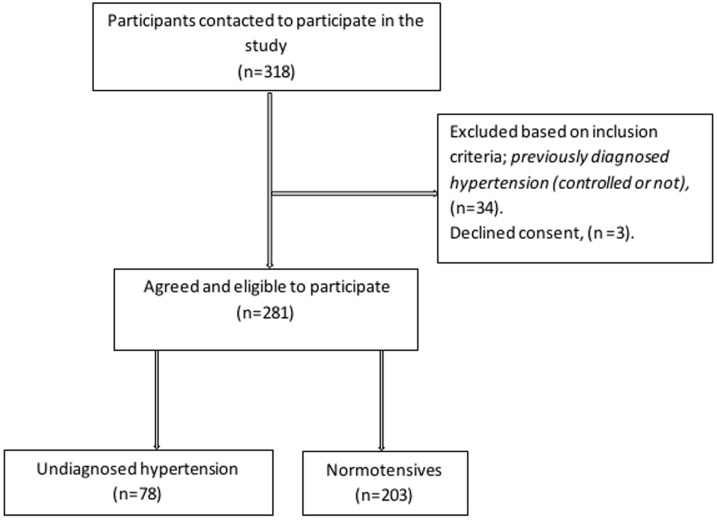
a flowchart showing the process of participants recruitment in the study

**General characteristics:** a total of 281 staff of Bauchi State University, Gadau participated in this study. The mean age of the participants was 34.5 ± 5.69 years, while their mean BMI was 23.1 ± 5.17 kg/m^2^. Majority of the participants were married (77.9%), and there were more males (56.2%) compared to female participants. A slight majority of the participants were academic or teaching staff (53.4%), while non-teaching staff accounted for the remaining participants. The mean systolic and diastolic blood pressure of the participants was 126.5 ± 13.39 and 82.8 ± 8.16 mmHg, respectively. The prevalence of undiagnosed hypertension was observed among 27.8% of the participants as indicated in Figure 1.

The results of the demographic and clinical variables based on the hypertensive status are presented in Table 1. Here, compared to the participants who were normotensives, those with undiagnosed hypertension were statistically older, taller, had less duration of working years (experience) and more body weight, and were more likely to be married (p<0.05). A sizeable number of the participants with undiagnosed hypertension classified themselves as being physically inactive or sedentary (82.1%) compared to the normotensive group (34.5%). The results also indicated both groups had a similar trend in terms of gender distribution and smoking status (P>0.05). Statistically significant differences in the trend of educational attainment, socioeconomic status and family history of hypertension were also revealed (Table 1).

**Table 1:** distribution of demographic and clinical characteristics of the participants based on hypertension status

Normotensive	Undiagnosed		
Variables	(n, 203)	Hypertension (n,78)	p-value
Age (years)	32.8 ± 3.72	38.9 ± 7.36	0.000^m^
**Gender**			**1.67^x^**
Male	109 (53.7)	49(62.8)	
Female	94 (46.3)	29(37.2)	
Height (m)	1.69 ± 0.07	1.73 ± 0.08	0.000^m^
Weight (kg)	63.4 ± 14.82	76.1 ± 15.42	0.000^m^
Work experience (years)	7.9 ± 3.92	7.4 ± 2.49	0.031m
**BMI (kg/m^2^)**			**0.000^x^**
Under-weight	85(41.9%)	13(16.7%)	
Normal weight	35(17.2%	14(17.9%)	
Over-weight	50(24.6%)	34(44.9%)	
Obese	33(16.3%)	16(20.5%)	
**Job cadre**			**0.001^x^**
Academic staff	96(47.3%)	24(30.8%)	
Non-academic staff	107(52.7%)	54(69.2%)	
**Smoking status**			**0.146^x^**
Yes	21(10.3%)	13(16.7%)	
No	182(89.7%)	65(83.3%)	
**Family history**			**0.000^x^**
Yes	144(70.9%)	37(47.4%)	
No	59(29.1%)	41(52.6%)	
**Marital status**			**0.000^x^**
Single	141(69.5%)	0(0%)	
Married	62(30.5%)	78(100%)	
Widowed/divorced	0(0%)	0(0%)	
**Educational attainment**			**0.000^x^**
Secondary	4(2%)	13(16.7%)	
Diploma (sub-degree)	91(44.9%)	26(33.3%)	
Bachelor's degree	1(0.5%)	15(19.2%)	
Master's degree	99(48.8%)	16(20.5%)	
Doctorate degree	8(3.9%)	8(10.3%)	
**Socioeconomic status**			**0.013^x^**
Low	21(10.3%)	7(9%)	
Moderate	169(83.3%)	57(73.1%)	
High	13(6.4%)	14(17.9%)	
**Physical activity level**			**0.000^x^**
Physically active	133(65.5%)	14(17.9%)	
Sedentary	70(34.5%)	64(82.1%)	

Notes: data are expressed as mean ± standard deviation for continuous variables and as absolute frequency and percentages for categorical variables; key: m: meters; kg: kilogram; n: number; *significant at 0.05 alpha level (bolded); ^m^=Mann Whitney U for comparison of independent samples: comparison of continuous non-normally distributed variables between normotensives and participants with undiagnosed hypertension; ^x^ =Chi square test: comparison of categorical variables between participants with undiagnosed hypertension and normotensives

**Prevalence and correlates of undiagnosed hypertension:** the prevalence of undiagnosed hypertension was observed among 27.8% of the participants. Following the univariable logistic regression analysis, all the independent variables of the study, i.e. age, gender, smoking status, family history of hypertension, job cadre, BMI, self-reported physical activity level and socioeconomic status were all found to be significantly associated with the dependent variable (undiagnosed hypertension), using a p-value cut off of <0.2 (for all). Therefore, all the parameters were included in the multivariate logistic regression analyses to determine the contribution of each of the variables to the model after controlling for the influence of others as presented in Table 2. The results indicated that participants with a positive family history of hypertension had an 83% odds of having undiagnosed hypertension than those without a family history (aOR: 0.833, 95%CI: 16.55-432.87, p= 0.000). Next, participants with a higher BMI score (aOR: 0.425, 95%CI: 0.085-0.447, p= 0.000), male gender (aOR: 0.451, 95%CI: 0.141-0.829, p= 0.018), (aOR: 0.515, 95%CI: 0.073-0.550, p= 0.002) and low physical activity level (aOR: 0.572, 95%CI: 5.296-49.777, p= 0.000) were also found to have a around half the odds for having undiagnosed hypertension. The smoking status and socioeconomic status of the participants were not significantly associated with undiagnosed hypertension (p<0.05) (Table 2).

**Table 2:** univariable and multivariable correlates of undiagnosed hypertension of the study participants

Independent variables	Univariate			Multivariate		
	OR	95%CI	p	aOR	95%CI	p
Age	0.048	0.697 - 0.841	0.000	0.089	0.647 - 0.916	0.003
Gender	0.273	0.402 - 1.173	0.168	0.451	0.141 - 0.829	0.018
Family history of hypertension	0.274	0.216 - 0.633	0.000	0.833	16.55 - 432.87	0.000
Smoking status	0.381	0.821 - 3.660	0.149	0.735	0.150 - 2.678	0.536
Job cadre	0.283	1.441 - 4.365	0.001	0.515	0.073 - 0.550	0.002
BMI	0.124	0.506 - 0.822	0.000	0.425	0.085 - 0.447	0.000
Physical activity level	0.330	0.060 - 0.220	0.000	0.572	5.296 - 49.777	0.000
Socio economic status	0.312	0.276 - 0.938	0.030	0.508	0.767 - 5.620	0.150

BMI: body mass index; OR: odds ratio; aOR: adjusted odds ratio; CI: confidence interval; P: alpha probability level (significant at 0.2)

## Discussion

This study aimed to examine the prevalence and correlates of undiagnosed hypertension among the staff of Bauchi State University, Gadau in Nigeria. Our major result indicated that the prevalence of undiagnosed elevated blood pressure (hypertension) was 27.8 percent among the population of the study. In addition, the current study had a relatively lower mean age, which makes it less likely for our participants to be diagnosed or have hypertension compared to previous studies that reported a higher prevalence [22,28,29].

In this study, blood pressure was classified according to the Seventh Joint National Committee criteria of systolic blood pressure of ≥140 mm Hg and/or diastolic blood pressure of ≥90 mm Hg [28], which is similar to the standards recommended by the European Society of Cardiology (ESC) and the European Society of Hypertension (ESH) classifications [1,30]. The findings of this current study are in line with studies in Nigeria. For example, Fatiu *et al*. (2003) reported the prevalence of hypertension in Nigeria to vary between 12% and 32.8% [29]. They further emphasized that a significant proportion of hypertensive subjects are unaware of their hypertensive status, which we have further highlighted in our study. Moreover, Egbi *et al*. (2015) has reported that BMI, long working years and advanced age are important correlates of undiagnosed hypertension [19]. Chronic hypertension and undiagnosed hypertension among Nigerians have also been associated with high rates of proteinuria and chronic kidney diseases among Nigerians in recent studies [29,31]. Therefore, there is a need to further identify potential preventive options.

Regarding specific population groups, Vincent-Onabanjo *et al*. (2017), reported that one-in-four traders of a regional market in Nigeria had undiagnosed hypertension [32], and similar results have been reported among community dwelling individuals in Southern Nigeria [33]. Studies among university workers in Nigeria have also indicated a high prevalence of hypertension [17,22]. All these results show a similar trend to the current study. Generally, we expected our study participants to be knowledgeable about their basic health indices such as their blood pressure status because they work in a university setting that is expected to offer a better advantage in terms of awareness compared to community-dwelling individuals in Nigeria. However, these results may not be surprising since a higher prevalence of hypertension and undiagnosed hypertension have been reported even among health workers in Nigeria [34]. This is an indication that elevated blood pressure values are mostly ignored and possibly underestimated by many Nigerians in their workplaces. Thus, the need for workplace strategies for increased awareness as a prevention and control strategy is imperative. Moreover, educational attainment or occupational status has not been proven to have a substantial influence on the attitude of individuals towards their state of health.

Our study also found that positive family history of hypertension, male gender, physical inactivity, lower job cadre, and high BMI scores to be variables with the most odds for undiagnosed hypertension. For family history, a positive history of hypertension from parents of relatives has been widely known to be a risk factor for hypertension. Moreover, family history is classified as a non-modifiable risk factor. The other findings are similar to the results of a recent study conducted in Sri Lanka [35]. Nevertheless, not all studies are in agreement with our study results. Another Sri Lankan study, unlike the findings of our study, reported physical activity level to be an insignificant predictor of undiagnosed hypertension [36].

The current study also had more males than females. Incidentally, gender was also found to be a significant correlate of undiagnosed hypertension in this study. Generally, males are known to have a relatively higher blood pressure than females [14]. Nevertheless, both males and females have been reported to have a poor perception of hypertension and awareness of lifestyle-modification measures in Nigeria [37]. Furthermore, factors such as marital status and educational attainment were not included in the multiple regression model due to the lack of representativeness of the data. For example in the undiagnosed hypertension group, all of the respondents indicated that they were married.

Additionally, our results are in conflict with part of the that of Kanungo *et al*. (2017), who reported significant predictors of undiagnosed hypertension to include being of younger age, poor education and lower socioeconomic status [38]. Our study results have highlighted that despite the relatively young age of the respondents, those with higher age within the cohort still showed a higher risk of being undiagnosed with hypertension. Therefore, this emphasizes the need for early screening and awareness campaigns on preventive measures in workplace in similar settings as earlier opined [39].

The study has a few limitations. We relied on the use of self-report for some of the parameters like physical activity level, which may introduce a possibility of recall bias. However, we do not think that the majority of the participants would fall into this category owing to their educational level and occupational status. Nevertheless, there may be a need for future studies using objective measures of physical activity like pedometers or accelerometers for assessing physical activity in similar settings in order to further validate our findings.

## Conclusion

A high percentage of the studied population have undiagnosed hypertension that is largely influenced by a non-modifiable such as a positive family history of hypertension, as well as modifiable (BMI scores, physical activity level and job cadre) risk factors. These variables can be used for early identification and in designing appropriate preventive strategies for populations in similar settings.

### What is known about this topic

- The prevalence of hypertension among various population groups in Nigeria is high, but variable;
- Already a number of variables such as age, body mass index, gender, and stressful working conditions have been reported to predispose to developing hypertension.

### What this study adds

- Having a positive family history of hypertension offers the highest chance of having undiagnosed hypertension in the studied population;
- The rate of undiagnosed hypertension in the studied population remains high and similar to other sub-population groups in Nigeria.
